# Quality of life and influencing factors of patients with rheumatoid arthritis in Northeast China

**DOI:** 10.1186/s12955-020-01355-7

**Published:** 2020-05-04

**Authors:** Bingqing Bai, Meng Chen, Lingyu Fu, Haina Liu, Lei Jin, Tingting Wei, Fangran Xin

**Affiliations:** 1 Department of Clinical Epidemiology and Evidence-based Medicine, the First Affiliated Hospital, China Medical University|, No.155, Nan Jing Bei Street, Shenyang, Liaoning Province China; 2 Department of Medical Record Management Center, the First Affiliated Hospital, China Medical University, Shenyang, China; 3 Department of Rheumatology, the First Affiliated Hospital, China Medical University, Shenyang, China; 4 Department of Rheumatology, Shengjing Hospital of China Medical University, Shenyang, China

**Keywords:** Rheumatoid arthritis, Quality of life, Risk factors, Health survey, Patient health questionnaire

## Abstract

**Purpose:**

Rheumatoid arthritis (RA) is a disease with a high disability rate, resulting in severe family and social burden. The aim of treatment is to improve the health-related quality of life (QoL) of patients. The purpose of this study was to evaluate the QoL of patients with RA in Northeast China and analyze its influencing factors.

**Methods:**

The study group consisted of 200 patients diagnosed with RA. The control group consisted of 200 healthy subjects. All subjects were residents in Northeast China. The investigation was conducted by questionnaire survey and electronic medical record. The WHOQOL-BREF, The Short-Form 36 Health Survey (SF-36) and Quality of Life Instruments for Chronic Diseases-RA (QLICD-RA) were used as questionnaires.

**Results:**

The QoL scores acquired by SF-36, WHOQOL-BREF and QLICD-RA scales showed significant differences between RA and control groups (*P* < 0.001). Multiple regression analysis showed that sleep duration (*P* = 0.001), psychological counseling (*P* < 0.001) and C4 level (*P* = 0.001) influenced the SF-36 scale evaluation model. IgA levels (*P* < 0.001) and being overweight (*P* = 0.030) were included in the WHOQOL-BREF evaluation model. Adequate sleep (*P* = 0.001) and psychological counseling(*P* = 0.050) entered the QLICD-RA scale evaluation model (*P* = 0.050), in which psychological counseling, normal C4 levels and being overweight were protective factors for RA, insufficient sleep and IgA levels were risk factors for RA.

**Conclusions:**

The QoL of RA patients is generally lower than those of healthy subjects in the Northeast China, Northeast China. Sleep duration, BMI (Body mass index), psychological counseling, C4 and IgA levels are factors that influence the QoL scores of RA patients.

## Introduction

Rheumatoid arthritis (RA) is a chronic inflammatory disease that causes pain and swelling of the joints, with disability rates up to 50%, seriously affecting the quality of life (QoL) of patients [1]. The prevalence rates of RA in China are 0.32% ~ 0.36%, whilst those in Northeast China are 0.5% [2], the highest prevalence rates in China. RA causes tremendous psychological pressure and long-term treatment costs impose huge economic burdens on RA patients^5^ and their families. The World Health Organization (WHO) defines QoL as “an individual’s perception of their position in life in the context of the culture and value systems in which they live and in relation to their goals, expectations, standards and concerns”.

In recent years, the QoL scale has been used in the evaluation of QoL and the selection of treatment options for normal and patient populations, effectively improving our understanding of disease and health [3, 4]. The measurement of QoL scores requires the selection of appropriate scales. To-date, scales at home and abroad possess a range of characteristics and application scopes [5–7]. Ding et *al.* [8] used SF-36, SAS and SDS scales to evaluate the QoL of RA patients in Beijing, whilst Chen et *al.* [9] used the QLICD-RA scale to evaluate the QoL of RA patients in Guangdong Province. However, there are no studies on QoL in patients with RA have been performed in Northeast China.

The WHOQOL-BREF [10] was simplified from WHOQOL-100 [11], both of WHOQOL-BREF and the SF-36 health survey have been widely used across the globe [12, 13]. However, which different is that QLICD-RA [14] was developed for chronic diseases through the combination of general and disease-specific modules by Chinese scholars. At this stage, the QLICD-RA scales showed high reliability, validity and sensitivity [15, 16]. This study is the first time to compare it with other two kinds of questionnaires, hoping to compare the reliability and validity of the three QoL scales.

This study systematically investigated the clinical data and demographic data of RA patients, and evaluated and compared the QoL of RA patients and healthy individuals using three QoL scales, respectively. We analyzed the influencing factors of the QoL of RA patients, to provide a platform to improve the QoL of RA patients.

## Methods

### Patient selection

In this study, we consecutively collected 200 RA patients from January 2017 to May 2018. 200 RA patients from the department of Rheumatology of the first affiliated hospital of China medical university were selected as the case group. The diagnostic standards for RA were revised by the American College of Rheumatology (ACR) in 1987 [17], as the sole diagnostic criterion for included patients. The control group consisted of 200 healthy subjects (normal neutrophils) from the physical examination center of the General Hospital of Northern Theater Command. All participants were residents in the Northeast China. Inclusion criteria: (1) adults (> 18 years old), ensure that there was no statistical difference in age and sex between the case group and the control group; (2) Educated to a level whereby the questionnaire can be completed; (3) No mental illness or disturbance of consciousness. Exclusion criteria: (1) autoimmune diseases other than RA or other joint diseases; (2) delirious and unconscious patients; (3) patients unwilling to cooperate; (4) pregnant or nursing women or women with pregnancy intention;(5) cancer patients whose life expectancy is less than 6 months;(6) patients with other serious illnesses that affect quality of life: cardiovascular diseases, cerebrovascular diseases, chronic respiratory diseases, diabetes, etc. All clinical information and laboratory indicators were approved by the participants, and informed consent was signed. Three investigators were trained in a unified manner.

### Data collection

The study participants were investigated by unified Chinese version of questionnaires. Investigations were performed using a questionnaire survey, and we collected information by electronic medical records.. During the investigation period, 20 investigators were randomly selected for secondary measurements for the assessment of retest reliability (retest interval > 1 week).

The three scales included: (1) WHOQOL-BREF: containing 26 items divided into four dimensions: (i) psychology, (ii) physiology, (iii) society and (iv) the environment;(2) SF-36: containing 36 items in 8 areas including physical health, physical function, physical enginery, body pain, energy, social function, emotional function and mental health; (3) QLICD-RA: divided into 4 dimensions, namely (i) physical; (ii) psychological; (iii) environmental; and (iv) RA, totaling 44 items. In these three questionnaires, the total score of each dimension is 100 points. According to the scoring rule of each questionnaire [18–20], the score of each item ranges from 1 to 5 points.

**The data to be collected included:**

1. Demographic data: name, gender, age, height, weight, education background, occupation, marital status, medical insurance and income.
2. Living and environmental factors: working environment, living conditions, transportation, eating habits and sleep duration.
3. Behavioral factors: smoking, drinking, long-term exercise and psychological counseling.
4. History of disease, allergy and hereditary disease history: hypertension and diabetes; allergies and hereditary history of RA.
5. Clinical biochemical indexes (obtained from the medical record information system): C reactive protein (CRP), complement C3, complement C4, immunoglobulin (IgG, IgA, IgM), anti-streptolysin O (ASO), erythrocyte sedimentation rate (ESR), rheumatoid factor (RF) and anti-cyclic peptide containing citrulline (anti-CCP).(only case group)

(6) QoL score scales: including WHOQOL-BREF, SF-36 and QLICD-RA scales;

here are Index measurement and definition, and scale scoring principles should See appendix.

### Index measurement and definition

1. Smoking: smoking ≥ 1 cigarettes per day for more than one consecutive year are defined as smokers; people who seldom smoke or stop smoking for more than a year are defined as non-smokers.
2. Alcohol: average daily consumption of 50 g of liquor or 1 bottle of beer with duration ≥ 1 year are defined as drinkers; People who seldom drink alcohol or have abstained from drinking for more than 1 year are defined as nondrinkers.
3. Long-term exercise: Take planned, purposeful physical activity performed with the intention of acquiring fitness or other health benefits. The number of times of exercise per week ≥ 3, and each time for more than 30 min are considered frequent exercisers; People who exercise less than 3 times a week or less than 30 min a week are considered infrequent exercisers [21].
4. Sleep duration: less than 5 h is insufficient sleep, 5 ~ 7 h is normal sleep, more than 7 h is sufficient sleep [22].
5. Psychological counseling: once or now have the experience of consulting or treating with a professional psychologist.
6. Working environment: extreme conditions such as noise or humidity are defined as special environment, otherwise normal environment.
7. Living conditions: If living house of the patient has only one floor, no stairs defined as flat floor; and apartment or non-residential floor defined as building.
8. Transportation: The definition of traffic trip is divided into automatic traffic and human traffic such as walking or cycling.

### Data analysis

Epidata was used for data entry. Statistical analyses were performed using IBM SPSS Statistics 22.0.(Statistical Product and Service Solutions, USA). For descriptive analysis, the enumeration data is expressed as a percentage n (%). Mean ± standard deviation was used to represent the measurement data conforming to the normal distribution. For statistical analysis, measurement data in the univariate analysis were statistically analyzed by *Stu*dent’s t test, F-test (joint hypotheses test) or rank sum test. Count data were analyzed by chi-square tests, and factors affecting the QoL scales were assessed by Multiple Linear Regression. Scores of each scale were converted into standard scores of the percentage system (S = (X-Min) × 100/R, S = standard score, X = major score, Min = minimum of the field score, R = the range of the field or total score). Probability testes were two-sided. Results were considered significant at *P* < 0.05.

The reliability of the scale was assessed using the Cronbach’s alpha as the internal consistency, and the reliability index of the re-test, the Cronbach’s alpha > 0.6 was used as the lowest criterion [23]. In the scale validity test, the exploratory factor analysis used the Kaiser-Meyer-Olkin (KMO) statistic > 0.70, and Bartlett test of Sphericity(*P* < 0.01) to suggest the conditions for factor analysis. Pearson’s correlation cofficient was used for scale correlation. The reliability test results of the three scales are shown in Table 1. SF-36 is acceptable, WHOQOL- bref is good, and QOLICD-RA is poor. As to the structural validity test results, all the three scales reached the validity test standards, their structural validity was good. The correlation test of the scale showed all three scales have good correlation.

**Table 1:** Scale effectiveness test

reliability	Internal consistency reliability	Retest reliability	validity
	α	α	
SF-36	0.69	0.72	acceptable
WHOQOL-bref	0.84	0.86	fair
QLICD-RA	0.42	0.69	poor
structure validity	KMO	Bartlett’s sphericity test	cumulative contribution
SF-36 scale	0.94	P < 0.001	80.23%
WHOQOL-bref	0.95	P < 0.001	80.23%
QLICD-RA	0.94	P < 0.001	70.24%
scale correlation	SF-36	WHOQOL-bref	QLICD-RA
	r *P*	r *P*	r *P*
SF-36	1 -	0.84 <0.001	0.92 <0.001
WHOQOL-bref		1 -	0.87 <0.001
QLICD-RA			1 -

*Reliability: Cronbach’s alpha; structure validity: Kaiser-Meyer-Olkin (KMO) statistic; Bartlett test of Sphericity; scale correlation: Pearson’s correlation coefficient

## Results

### Comparison of QoL between RA patients and healthy controls

**Fig. 1:**
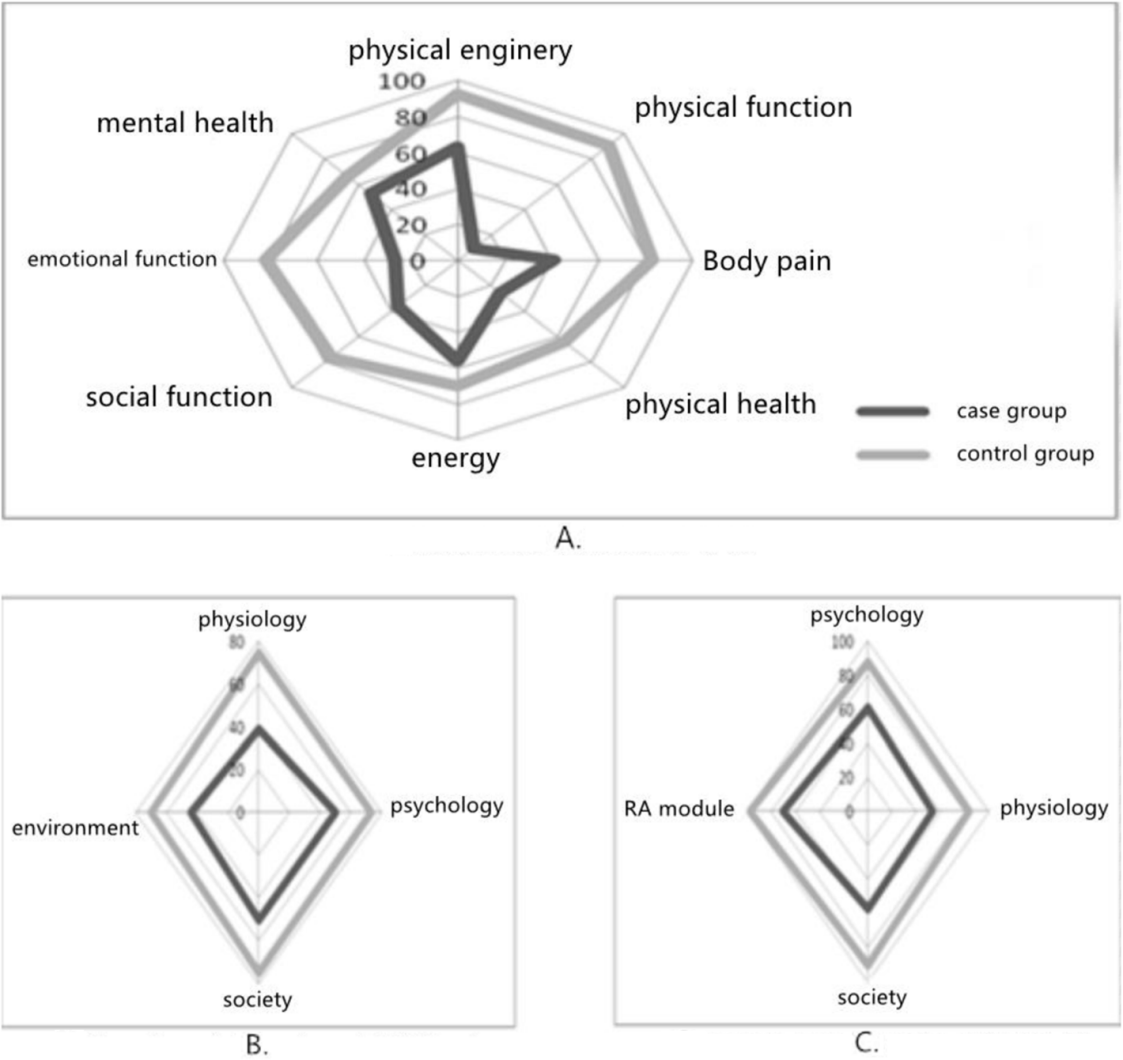
Comparison of QoL between RA patients and healthy controls. **a** SF-36 scale. Score in each dimension of SF-36. **b** WHO-bref scale. Score in each dimension of WHO-bref. **c** QLICD-RA scale. Score in each dimension of QLICD-RA

The total scores of the three scales and QoL scores of each dimension statistically differed between RA and control groups (*P* < 0.010). The QoL scores of the RA group were generally lower than those of the healthy control group (Fig. 1). The radar map showed that the SF-36 scale (Fig. 1a) more sensitively displays the changes in the QoL of RA patients. In its eight dimensions, the physiological functions and dimensions of the RA group had the lowest values, with energy and mental health scores in the RA group comparable to control groups. Amongst the four dimensions of WHOQOL-BREF (Fig. 1b) and QLICD-RA (Fig. 1c), the scores between the RA group and healthy group differed to comparable levels across the dimensions.

### Analysis of factors affecting the QoL of RA patients

Using the SF-36, WHOQOL-BREF, and QLICD-RA scale, factors that resulted in significant differences in the QoL scores of RA patients (Table 2) included: age, BMI index, marriage, sleep duration, psychological counseling, CRP, C4, IgA and IgM levels. The QoL scores of overweight patients were highest in weight groups; patients younger than 50 had higher QoL scores than those older than 50. Singlehood and psychological counseling experiences were protective factors of the QoL scores, whilst insufficient sleep, abnormally elevated CRP, C4, IgA and IgM levels were risk factors for the QoL scores of RA patients.

**Table 2:** Comparison of QoL scores in RA group

Variables		mean ± SD	*P*
SF-36			
BMI	Thin	24.24 ± 22.77	
	normal	37.43 ± 15.21	0.001
	overweight	40.66 ± 15.21	
Sleep duration	Normal	38.21 ± 13.80	
	Adequate	43.02 ± 17.75	
	Insufficient	23.23 ± 11.51	0.001
Psychological counseling	Yes	55.70 ± 17.03	
	No	38.60 ± 15.04	<0.050
C4	Low	35.89 ± 20.68	
	Normal	40.62 ± 14.16	
	High	24.79 ± 21.36	0.020
WHOQOL-bref			
BMI	Thin	41.33 ± 12.52	
	normal	43.70 ± 7.47	
	overweight	47.06 ± 9.56	<0.001
IgA	Low	49.54 ± 6.77	
	Normal	47.91 ± 10.03	
	High	33.36 ± 7.74	<0.001
QLICD-RA			
Age	≥50y	59.43 ± 10.10	
	<50y	64.02 ± 11.89	0.001
BMI	Thin	48.67 ± 15.81	
	normal	59.26 ± 9.59	
	overweight	61.96 ± 10.32	<0.001
Marriage	Single	69.58 ± 13.95	
	Married	60.19 ± 10.41	0.030
Sleep duration	Normal	60.29 ± 9.66	
	Adequate	60.64 ± 12.38	
	Insufficient	49.22 ± 9.95	<0.001
Psychological counseling	Yes	72.55 ± 13.74	
	No	60.28 ± 10.49	<0.050
CRP	+	59.55 ± 11.48	
	–	63.36 ± 7.52	0.020
C4	Low	56.57 ± 17.71	
	Normal	61.89 ± 9.48	
	High	50.26 ± 14.71	<0.001
IgA	Low	64.24 ± 10.45	
	Normal	62.47 ± 10.10	
	High	57.79 ± 10.78	0.020
IgM	Low	66.40 ± 9.75	
	Normal	60.35 ± 9.75	
	High	58.47 ± 12.93	0.020

*Student’s t test; *n* = 200

### Multiple regression analysis of factors influencing the QoL in the RA group

Multivariate linear regression was performed on the total scores of the three scales. All significant variables in Table 2 were entered into the multiple regression analysis of the above three scales, and stepwise regression methods were used for analysis.

In the SF-36 scale’s final model, insufficient sleep (*P* = 0.001), psychological counseling (*P* < 0.001), and normal C4 levels (*P* = 0.001) entered the final model (Table 3); IgA elevation (*P* < 0.001) and being overweight (*P* = 0.030) entered the final model when the WHOQOL-BREF scale was employed. When the QLICD-RA scale was used for assessments, adequate sleep (*P* < 0.001) and psychological counseling (*P* = 0.050) entered the final model, amongst which psychological counseling, normal C4 levels and being overweight were protective factors of RA QoL. Insufficient sleep and elevated IgA levels were risk factors for RA QoL.

**Table 3:** Multiple linear regression of factors on influencing RA quality of life

Factor	B	SE	95%CI	β	t	*P*
SF-36						
Insufficient sleep	−5.48	1.26	−0.99 ~ − 7.95	−0.21	−2.62	0.001
Psychological guidance	26.76	8.49	43.4 ~ 10.12	0.22	2.75	<0.001
C4 normal level	7.23	2.51	12.15 ~ 7.23	0.21	2.62	0.001
ε	57.75	18.33	93.68 ~ 21.82	–	5.02	<0.001
WHOQOL-bref						
IgA evaluation	−4.39	1.57	−7.47 ~ −4.39	− 0.17	−2.25	<0.001
Overweight	1.12	0.41	0.32 ~ 1.12	0.52	2.85	0.030
ε	47.91	15.88	16.79 ~ 47.91	–	9.14	<0.001
QLICD-RA						
Adequate sleep	13.18	3.15	7.21 ~ 19.55	0.36	2.56	0.001
Psychological counseling	11.67	2.93	5.93 ~ 17.41	0.28	2.03	0.050
ε	74.06	23.64	51.36 ~ 96.76	–	5.2	<0.001

*Multiple Linear Regression; *n* = 200

### Analysis of factors influencing the QoL scores of RA patients in different dimensions

The radar diagram of the SF-36 scale showed that the lowest values occurred in the physical functions of the case group. The SF-36 scale was further divided into the general assessment of physical health (physical enginery, physical function, body pain, physical health) and mental health (energy, social function, emotional function, mental health) to analyze the influencing factors, respectively. The results showed (Table 4) that factors influencing the QoL scores of RA patients included: BMI (*P* = 0.030), transportation (*P* = 0.020), sleep duration *(P <* 0.001), psychological counseling (*P* < 0.050), ESR (*P* = 0.040), C4 (*P* = 0.040) and IgA (*P* = 0.030) levels. Overweight, automated vehicles and psychological counseling experiences were factors that increased the QoL score. Insufficient sleep, being ESR-positive, C4-positive and IgA-positive reduced the QoL score.

**Table 4:** Comparison of QoL scores of RA group based on SF-36 scale

	Variables		mean ± SD	*P*
physical health	BMI	Thin	20.00 ± 24.26	
		Normal	34.93 ± 15.89	
		Overweight	35.90 ± 14.12	0.030
	Transportation	Walking/Cycling	31.80 ± 15.44	
		Automated vehicle	38.81 ± 15.04	0.020
	Sleep duration	Normal	38.83 ± 19.28	
		Adequate	34.14 ± 14.60	
		Insufficient	17.75 ± 10.44	<0.001
	ESR	+	29.74 ± 12.94	
		–	38.81 ± 14.67	0.040
	C4	Low	31.63 ± 22.01	
		Normal	36.50 ± 15.13	
		High	21.18 ± 22.19	0.040
	IgA	Low	32.38 ± 6.17	
		Normal	37.55 ± 16.19	
		High	30.96 ± 14.56	0.030
mental health	Sleep duration	Normal	47.21 ± 18.17	
		Adequate	42.27 ± 14.26	
		Insufficient	28.70 ± 12.87	<0.001
	Psychological counseling	Yes	65.49 ± 18.59	
		No	42.76 ± 15.45	<0.050

*Student’s t test; *n* = 200

Further multiple regression results are shown in Table 5. In the evaluation of physical health dimensions, insufficient sleep (*P* = 0.030) and automated vehicles (*P* = 0.040) entered the final model. Patients with sleep insufficiency had lower QoL scores. The use of autonomous vehicles increased the QoL of RA patients. In overall mental health evaluation dimensions, normal sleep (*P* = 0.020), adequate sleep (*P* = 0.001) and psychological counseling (*P* < 0.050) entered the final model. Regarding mental health scores, patients with normal and adequate sleep scored higher, as did patients with psychological counseling experience.

**Table 5:** SF-36 multivariate regression of QoL influencing factors of physical and mental dimensions

Factor	B	SE	95%CI	β	t	*P*
physical health						
Insufficient sleep	−5.3	1.32	−7.89 ~ −2.71	−0.16	−2.21	0.030
Automated vehicle	1.83	0.61	0.63 ~ 3.03	0.08	1.06	0.040
ε	36.01	9.77	16.86 ~ 55.16	–	5.47	<0.001
mental health						
Normal sleep	4.83	1.28	2.33 ~ 7.33	0.17	2.37	0.020
Adequate sleep	13.34	3.02	7.42 ~ 19.26	0.19	2.55	0.001
Psychological counseling	15.83	4.13	7.74 ~ 23.52	0.12	1.78	<0.050
ε	57.88	13.59	31.24 ~ 84.52	–	6.57	<0.001

*Multiple Linear Regression; *n* = 200

## Discussion

To our knowledge, despite its highest incidence of RA, this is the first study to assess the QoL of RA patients in Northeastern China. We employed 3 scales, the WHOQOL-BREF, SF-36 and QLICD-RA, to assess the quality of RA patients, respectively. All three scales reached the validity test standards, structural validity was good and the scales showed high levels of correlation. This study demonstrated a significant difference of the QoL levels between RA patients and healthy individuals, indicating that the ability of RA patients to live and work were seriously affected by the disease. Sleep duration, BMI, psychological counseling, C4 and IgA levels could affect the QoL of RA patients. Dimensional analysis of the influencing factors based on the SF-36 scale showed that sleeping and transportation affected the physical health scores of RA patients, whilst sleeping and psychological counseling affected the mental health scores of RA patients. These findings highlight methods to improve the QoL of patients.

Sleeping can affect the QoL. To some extent, sleep duration and quality can reflect the QoL of ordinary individuals and patients. Luyster et al. [24] showed that 54–70% of patients had sleeping problems, including difficulties falling asleep, poor sleep quality and daytime sleepiness. In this study, the QoL of RA patients increased with increased sleep duration. The reasons were that RA sleep duration may vary depending on the severity of the disease. As pain is a major symptom, RA is likely to affect patients’ sleep duration. Studies have shown that long-term pain [25], fatigue, joint swelling, and disease activity in RA patients [26] are factors influencing sleep disorders. Wolfe and Xu et al. [27, 28] proposed that both pain and psychological depression were independent risk factors for RA sleep disorders.

With the gradual development of psychological medicine, clinical and scientific researchers have paid increasing attention to the psychosomatic and emotional health of RA patients. Domestic and foreign studies have shown that depression, anxiety, irritability and other adverse emotions are more serious in RA patients [29]. These negative emotions can affect various systems throughout the body through a variety of factors, such as the endocrine system and nervous system, and can affect the immune system, leading to the aggravation of disease [30]. Similarly, RA has a heavy disease burden, and the perennial pressure of physical, mental and economic burdens can affect treatment compliance, resulting in a loss of QoL. In this study, although formal psychotherapy is not common in China, the QoL of patients receiving psychological counseling was higher than those not receiving counseling, highlighting its ability to alleviate low QoL in RA patients, consistent with previous findings [31, 32]. a comprehensive improvement in both sleep and patients’ psychological state is therefore of great significance to the improvement of QoL.

To-date, the association between obesity and RA is uncertain, but there is evidence that sex hormones influence RA incidence, and that overweight and obese women have an increased risk of RA [33]. In addition, a large number of fat cells can influence immune function and promote inflammation. In severe cases, the metabolic syndrome and other diseases contribute to the increased risk of RA [34]. In contrast, studies have shown that muscle protein loss in RA patients leads to a decrease in BMI and QoL [35], consequently, high BMI levels are protective factors for bone destruction [36]. The appropriate increase in BMI was beneficial to improve the QoL of patients in this study. It has been suggested [37] this occurs as RA cachexia has not yet appeared, and that the BMI can reflect the degree of obesity in patients. A prolonged disease course leads to increased muscle protein decomposition, which ultimately leads to decreases in the BMI. In this case, the BMI did not reflect patient obesity objectively. The climate in Northeast China is cold and the high BMI rates of patients with RA indicates they have sufficient calories for cold resistance. However, no consensus has been reached, and the specific reasons require further exploration.

IgA and C4 levels are important auxiliary diagnostic biological indicators for RA, and their activity can indicate disease severity. As an immunoglobulin, IgA is associated with immune function. When inflammation or tissue damage occurs in the human body, the severity of RA is often determined by the comprehensive evaluation of these indicators. Clinical studies have shown that IgA levels change according to several inflammatory specific indicators. Others have shown that IgA levels positively correlate with the DAS28 score [38], suggesting that IgA levels have reference values for the determination of RA disease activity. Complement C3 and C4 exist in the healthy human body, and blood complement C3 and C4 levels directly change according to various inflammatory diseases in the body. The dynamic observation of complement levels is conducive to auxiliary observations of RA clinical conditions.

This study also showed that patients who use automated vehicles have a better QoL. In the SF-36 scale, the lowest physical function values were in the RA group (representing patients with RA that subjectively believe that the disease seriously affects their ability to work and live). The possible reasons for this are that most RA patients frequently use cars, subways, buses and other autonomous vehicles due to their disability. In Northeast China, the winter is also long and cold, so automated vehicles can greatly reduce the mobility difficulties of RA patients.

To our knowledge, there is still no internationally recognized QoL scale for RA. We selected three relevant scales and highlight that the SF-36 scale is flexible, and applicable to the Chinese population [39] and shows high reliability and validity [40, 41].

WHOQOL-BREF is a comprehensive evaluation scale for patients’ QoL that is characterized by a wide application range [42], strong cross-cultural adaptability, and can be used to compare patients with RA in different stages [43]. The QLICD-RA scale shows good consistency for RA QoL assessments [15, 44]. The results of this study also highlight the applicability of QLICD-RA. The SF-36 scale reflects the influence of RA on the QoL more sensitively. Both WHOQOL-BREF and QLICD-RA have four dimensions that reflect changes in the QoL of RA patients from different aspects, with sensitivities lower than those of the SF-36. The possible reasons for the poor internal consistency of the QLICD-RA scale were investigation bias, group specificity, and physiological modules in the scale describing the QoL of RA patients from the perspective of physical health. If the results of the two areas of the scale are inconsistent, deviation is often observed.

## Limitations

The following limitations must be considered. Firstly, the sample source was small and all RA patients were from the department of rheumatology from the first affiliated hospital of China medical university. Recall bias may therefore have occurred. Some of the qualitative data of the variables (such as smoking) could also not be analyzed for quantitative correlations. Further studies are required to verify these results.

## Conclusions

In summary, the SF-36, WHOQOL-BREF and QLICD-RA show high correlation in measuring the QoL of RA patients. Our data showed that the QoL of RA patients was generally lower than that of healthy individuals in the Northeast China. In addition, sleep duration, BMI, psychological counseling, C4 and IgA may be influential factors for the QoL of RA patients in Northern China. Active measures to regulate sleep cycles, control C4, IgA and other indicators, together with appropriate psychological counseling may effectively improve the QoL of RA patients.

## Appendix

(1) Scoring Principles of the SF-36 Scale.

1. Dimensional Composition

Physiological function: questions 3-12 Physiological function: questions 13-16 Physical pain: questions 21-22 General health: questions1, 33-36 Energy: questions 23, 27, 29, 31 Social function: 20, 32 questions Emotional Functions: questions 17-19 Mental Health: 24-26, 28 and 30 Questions

2. Scoring

Positive scoring: questions 2-19, 24, 25, 28, 29, 31-33, 35

Reverse scoring: 1, 20-23, 26, 27, 30, 34, 36 questions

(2) Scoring Principles of the WHOQOL-BREF Scale

1. Dimensional Composition

Physiological Dimensions: 3, 4, 10, 15-18 Psychological Dimensions: 5, 6, 7, 11, 19, 26 Social Dimensions: 20-22 Environmental Dimensions: 8, 9, 12-14, 23-25

2. Scoring Direction

Positive Scoring: 1, 2, 5-25 Questions

Reverse Scoring: Questions 3, 4 and 26

(3) Scoring Principles of the QLICD-RA Scale

1. Dimensional Composition

Physiological Dimension: GPH1 ~ GPH10 Psychological Dimension: GPS1 ~ GPS11 Social Dimension: GS1-GS8 RA Block: RA1-RA15

2. Scoring Direction

Forward scoring: GPH1, GPH2, GPH4-GPH8, GPS1, GPS3, GPS10, GS1-GS5, GS8, RA1-RA15 Reverse scoring: GPH3, GPH9, GPH10, GPS2, GPS4-GPS9, GPS11, GS6, GS7

(4) Conversion of Percentage System in all dimensions

$$ Converted\ score=\frac{Actual\ score- the\ lowest\ possible\ score\ in\ this\ area}{The\ difference\ between\ the\ highest\ and\ the\ lowest\ possible\ score}\times 100 $$

## Abbreviations

ACR: American College of Rheumatology
anti-CCP: anti-cyclic peptide containing citrulline
ASO: anti-streptolysin O
BMI: Body mass index
ESR: erythrocyte sedimentation rate
KMO: Kaiser-Meyer-Olkin
QoL: quality of life
QLICD-RA: Quality of Life Instruments for Chronic Diseases-RA
RA: Rheumatoid arthritis
RF: rheumatoid factor
SF-36: Short-Form 36 Health Survey
WHO: World Health Organization
